# The Potential Usefulness of the Expanded Carrier Screening to Identify Hereditary Genetic Diseases: A Case Report from Real-World Data

**DOI:** 10.3390/genes14081651

**Published:** 2023-08-19

**Authors:** Iolanda Veneruso, Annaluisa Ranieri, Noemi Falcone, Lorella Tripodi, Carmela Scarano, Ilaria La Monica, Lucio Pastore, Barbara Lombardo, Valeria D’Argenio

**Affiliations:** 1 CEINGE-Biotecnologie Avanzate Franco Salvatore, via G. Salvatore 486, 80145 Naples, Italy; 2 Department of Molecular Medicine and Medical Biotechnologies, Federico II University, via Sergio Pansini 5, 80131 Naples, Italy; 3 Department of Human Sciences and Quality of Life Promotion, San Raffaele Open University, via di Val Cannuta 247, 00166 Rome, Italy

**Keywords:** expanded carrier screening, NGS, whole-exome sequencing, aCGH, autism spectrum disorder, prenatal testing

## Abstract

Expanded carrier screening (ECS) means a comprehensive genetic analysis to evaluate an individual’s carrier status. ECS is becoming more frequently used, thanks to the availability of techniques such as next generation sequencing (NGS) and array comparative genomic hybridization (aCGH), allowing for extensive genome-scale analyses. Here, we report the case of a couple who underwent ECS for a case of autism spectrum disorder in the male partner family. aCGH and whole-exome sequencing (WES) were performed in the couple. aCGH analysis identified in the female partner two deletions involving genes associated to behavioral and neurodevelopment disorders. No clinically relevant alterations were identified in the husband. Interestingly, WES analysis identified in the male partner a pathogenic variant in the *LPL* gene that is emerging as a novel candidate gene for autism. This case shows that ECS may be useful in clinical contexts, especially when both the partners are analyzed before conception, thus allowing the estimation of their risk to transmit an inherited condition. On the other side, there are several concerns related to possible incidental findings and difficult-to-interpret results. Once these limits are defined by the establishment of specific guidelines, ECS may have a greater diffusion.

## 1. Introduction

Expanded carrier screening (ECS), i.e., the simultaneous and extensive analysis of several disease-related genes to evaluate an individual carrier status, is becoming an even more common practice in clinical contexts [1]. Indeed, thanks to the availability of genome-wide technologies, such as next generation sequencing (NGS) and array comparative genomic hybridization (aCGH), it is possible to investigate multiple genomic loci with high accuracy and in a time- and cost-affordable manner [2,3,4]. As already reviewed elsewhere [1], with respect to traditional, more-focused approaches, ECS presents several advantages. Indeed, it is not affected by ethnicity, allows for identification of all possible disease-related DNA variants (including rare and novel variants), analyzes also genes considered uncommon for a specific phenotype, and includes the diagnosis of a high number of diseases [5,6]. These benefits are higher when both partners of a couple undergo ECS, thus allowing for an accurate estimation of their own couple’s risk of having a baby affected by a genetic inherited disease [7]. If ECS is performed before conception, this will allow them to plan the best reproductive choices based on the estimated risk [8]. It has to be underlined that, despite the above-mentioned advantages, several concerns still exist, such as the number of diseases or genes to be analyzed [9,10]. In particular, the implementation of genome wide analyses for ECS purposes, such as aCGH and NGS-based approaches, including targeted panels, whole-exome sequencing (WES), and whole genome sequencing, is focusing attention on the need for clear guidelines regarding both the genomic regions to be analyzed and the most proper methodological strategy to be used. It is well known that the more genomic data are obtained, the more their interpretation is challenging [11]. Indeed, these genomic tests have a high risk of generating results that are difficult to interpret and variants of uncertain significance (VUS), thus making communication and clinical management of the data obtained difficult.

As a case-study to exemplify the potential usefulness of ECS in clinical contexts, but also to highlight the limitations of this procedure, here we report the results obtained by analyzing a couple that performed ECS based on aCGH and WES analyses.

## 2. Case Presentation

**Figure 1:**
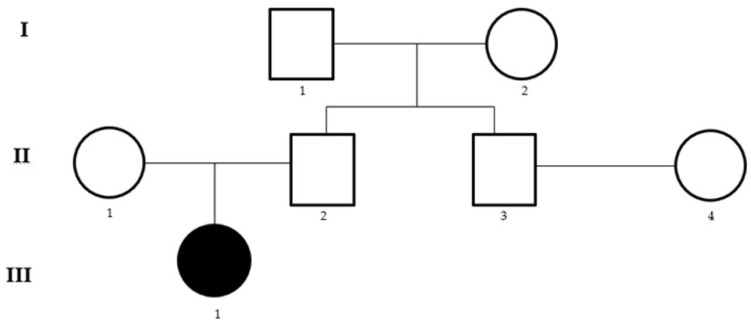
Pedigree of the studied family. Roman numbers indicate the different generations while the Arabic numbers the individuals within each generation.

ECS, by using both aCGH and NGS analyses, was performed in a couple (II.3 and II.4) whose male partner reported a case of autism spectrum disorder (ASD, III.1) in his family (Figure 1).

The patient (III.1, now 8-year-old) was born at term of an uncomplicated pregnancy to healthy non-consanguineous parents and was diagnosed as ASD based on her clinical phenotype; indeed, she has borderline intellectual functioning, emotional-behavioral regulation disorder, and mild dysmorphic notes. Unfortunately, no further information on the clinical history is available due to poor cooperation from her parents. In addition, this subject (III.1) and her parents (II.1 and II.2) were not available for molecular genetic tests. Therefore, there was no genetic information about the patient with ASD that could allow for a more focused estimation of ASD risk within the family.

After obtaining written informed consent from each individual of the couple, a peripheral blood EDTA sample was collected to carry out aCGH and WES molecular analyses. DNA extraction from peripheral blood was performed using the Maxwell RSC Blood DNA kit (Promega, Madison, WI, USA), according to manufacturer’s instructions. aCGH was performed by analyzing patients’ DNA with the 4 × 180 K SurePrint G3 Human CGH Microarray (Agilent Technologies, Santa Clara, CA, USA), according to the manufacturer’s directions [12]. Genomic positions were defined using NCBI38/hg38. This platform includes 180,000 60-mer oligonucleotide probes with an overall median probe spacing of 13 Kb; so that it allows an average resolution of 25 kb. Microarrays were analyzed using an Agilent G2600D scanner. Image files were quantified and data were visualized using Agilent’s Cytogenomics software (V.4.0.3.12). Analysis of the copy number variations (CNVs) contained in the interval-based report generated by Agilent’s Cytogenomics software was performed using Alissa bioinformatic software (Agilent) consulting Clinvar (https://www.ncbi.nlm.nih.gov/clinvar/, accessed on 29 June 2023), Decipher (https://decipher.sanger.ac.uk/, accessed on 29 June 2023), the Database of Genomic Variants (http://dgv.tcag.ca/dgv/app/home, accessed on 29 June 2023), GeneCards (http://www.genecards.org/, accessed on 29 June 2023), OMIM (https://www.omim.org/, accessed on 29 June 2023) and SFARI (https://gene.sfari.org/, accessed on 29 June 2023). Similarly for WES, starting from DNA samples, a sequencing library was prepared for both individuals by using the Human All Exon V7 targeted SureSelect XT HS enrichment system (Agilent Technologies, Santa Clara, CA, USA), according to manufacturer’s instructions, and as previously reported [13]. Sequencing reactions were performed using a Mid Output flow cell v2.5 (300 cycles) on the NextSeq 500 instrument (Illumina, San Diego, CA, USA). FASTQ files were imported and analyzed by using the Alissa bioinformatic software (Agilent). This web-based tool enabled us to perform both primary and secondary NGS data analysis, thanks to the possibility of integrating two different and sequential pipelines. Firstly, the Align and Call pipeline allowed us to map FASTQ data against the reference human genome sequence. Subsequently, the importing of the obtained vcf files/sample in the Interpret module gave us the capability to apply a prioritization tree that led to the identification of the clinically interesting variants. This software lacked specific commands that could allow for the matching of variants found in these two individuals. For this reason, all of the prioritized variants searched on dbSNP (https://www.ncbi.nlm.nih.gov/snp/, accessed on 29 June 2023) and ClinVar (https://www.ncbi.nlm.nih.gov/clinvar/, accessed on 29 June 2023) databases were manually matched. A further analysis of pathogenicity for each variant was assessed using the Varsome tool (https://varsome.com, accessed on 29 June 2023).

**Figure 2:**
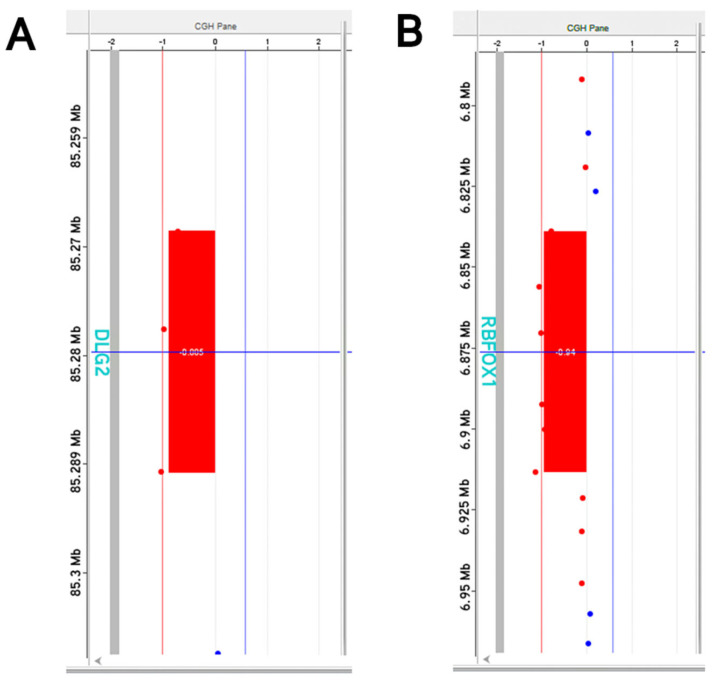
aCGH analysis results identified in the female partner of the analyzed couple. (**A**) aCGH profile of chromosome 11. This analysis shows a heterozygous deletion in 11q14.1 region of 22.4 kb, partially involving the *DLG2* gene. (**B**) aCGH profile of chromosome 16. The analysis shows a heterozygous deletion in 16q13.3 region of 74.8 kb, involving the *RBFOX1* gene. Results are interpreted as log2 ratio of test vs. control. Deletions are indicated by a red rectangle.

The aCGH analysis in the wife showed the presence of a heterozygous deletion on chromosome 11, in the q14.1 region, ranging from position 85,268,669 to 85,291,041, with an extension of 22.4 kb, partially involving the discs large MAGUK scaffold protein 2 (*DLG2*) (Refseq # NC_000011.10) gene (Figure 2A). Additionally, the analysis showed the presence of a heterozygous deletion on chromosome 16, in the q13.3 region, ranging from position 6,839,407 to 6,914,190, with an extension of 74.8 kb, involving the RNA binding fox-1 homolog 1 (*RBFOX1*) (Refseq # NC_000016.10) gene (Figure 2B). The aCGH analysis in the husband did not identify the presence of significant alterations.

Raw data obtained by WES molecular analysis in each sequenced sample are reported in Table 1.

The prioritization pipeline led to the identification of 61 variants for the man and 50 variants for the woman. These variants were individually checked on both dbSNP and ClinVar databases and evaluated for pathogenicity prediction using the Varsome platform. In this way we have identified four variants present in both the female and male partners of the couple (Table 2).

Moreover, three pathogenic/likely pathogenic variants were identified in the husband (Table 3). No other potentially clinically interesting variants were identified in the wife.

## 3. Discussion

The recent diffusion of ECS using advanced molecular techniques, allowing for the simultaneous analysis of several mutations or genes associated with different pathological conditions, makes it possible to evaluate the carrier status within a couple and to obtain a more accurate estimate of the genetic risk to conceive an affected baby. In our study, through the use of aCGH and WES, we performed ECS in a couple whose male partner reported a case of ASD in his family in order to identify genetic variants which, if transmitted, could represent an important risk factor for autism in offspring.

The aCGH analysis performed in this couple revealed the presence of two heterozygous deletions of uncertain clinical significance, only in the wife. In particular, the first deletion includes the *DLG2* gene, located on chromosome 11, cytoband q14.1 (83,455,012-85,628,373 (GRCh38/hg38), characterized by 23 exons for a total length of about 2,173,362 bases, with 50 transcripts, 219 orthologues and 3 paralogues. This gene encodes a member of the membrane-associated guanylate kinase protein superfamily of scaffold proteins, a component of the post-synaptic density in excitatory neurons and regulator of synaptic function and plasticity [14,15,16,17]. DLG2 protein has been reported as highly expressed in different adult rodent brain areas, including cortex, hippocampus, striatum and cerebellum [15,16]. Studies conducted on mouse models have shown that deletions of the *DLG2* gene lead to atypical locomotor responses, a reduced social approach and an increase in repetitive behaviors, highlighting that *DLG2* is involved in excitatory synaptic transmission [18,19]. In humans, *DLG2* mutations have been suggested as emerging genetic risk factor for several neurodevelopmental psychiatric disorders, such as schizophrenia, ASD, bipolar disorder [20,21,22], and intellectual disability (ID) [23]. In particular, de novo mutations causing *DLG2* loss of function have been described several times in schizophrenic patients [22,24] and *DLG2*-containing CNVs have been reported in patients with ASD [25,26]. Most of the genetic deletions observed in humans are heterozygous [23] and are present in male individuals [21,27]. In most cases, the alteration is inherited from the asymptomatic mother; this supports the female protective hypothesis for neurodevelopmental disorders, suggesting that the clinical manifestations of neurodevelopmental disorders require a higher “mutational burden” for females [23,28]. In addition to being more aberrant in males, transmission of the *DLG2* deletion from clinically asymptomatic parents suggests incomplete penetrance of the alteration [28]. The other alteration observed concerns the *RBFOX1* gene, which is located on chromosome 16, cytoband p13.3 (5,239,738-7,713,340 (GRCh38/hg38), and includes 25 exons for a total length of about 2,473,603 bases, with 40 transcripts and 178 orthologues. *RBFOX1* is a dose-sensitive gene that encodes splicing factors specifically expressed in neurons and muscles and is an important regulator of development and neuronal excitability [29,30,31]. Reduced *RBFOX1* expression has been associated with neurodevelopmental disorders; indeed, CNVs comprising *RBFOX1* are implicated in mental retardation, epilepsy, schizoaffective disorder (SCZ) [29,32], bipolar disorder (BD), attention deficit hyperactivity disorder (ADHD), ID [33] and ASD [34]. In mouse models, specific deletions of *RBFOX1* cause pronounced downregulation of *RBFOX1* resulting in hyperactivity, stereotyped behavior, impaired acquisition and reduced social interest [32,35]. The DECIPHER database shows that 82% of deletions in the *RBFOX1* gene are inherited from unaffected parents, underlining that affected individuals show a different phenotype than carrier parents [30,36]. This supports the hypothesis that there is a variable expressivity linked to the *RBFOX1* gene [29]. Although most of the molecular genetic variants strongly associated with neurodevelopmental disorders, particularly with ASD, are de novo, also variants inherited from unaffected parent can contribute to ASD [37].

The analysis of variants found after WES highlighted the presence of four variants that are present in both the husband and the wife (Table 2). Among them, the variant in the Methylenetetrahydrofolate reductase (*MTHFR)* gene is a common polymorphism associated to an increased risk of hyperhomocysteinemia, that is itself a risk factor for different cardiovascular diseases. This variant is considerably associated to ASD. Indeed, *MTHFR* gene encodes for a thermolabile enzyme that is less active at high temperature. Consequently, in presence of fever, cognitive and behavioral skills are impaired in those variant’s carriers by the hyperhomocysteinemia that modulates N-methyl-D-aspartate receptor activity. Additionally, *MTHFR* variant c.788C > T p.(Ala263Val) has been already suggested as a risk factor for schizophrenia development and evidences have been emerging about the possibility that schizophrenia and ASD share several risk-genes [13]. The c.47C > T p.(Ala16Val) variant in the *PRSS1* gene is associated to hereditary pancreatitis. Functional studies demonstrated that this variant has a damaging effect on the protein [38]. However, although it is characterized by an autosomal dominant inheritance, the presence of asymptomatic carriers, associated to its high allelic frequency in the general population, suggests for a reduced penetrance of this variant. Accordingly, ACMG criteria classify this variant as benign. Finally, the other two variants identified in both the partners of this couple are currently classified as VUS/conflicting and, to date, they seem to be not related to the pathological conditions that induced the request of this ECS. Interestingly, looking to individual variants present in each of the analyzed subjects, while in the wife we did not find any additional variants with potentially interesting clinical significance, in the husband we found three likely pathogenic/pathogenic variants (Table 3), one of which could be considered interesting in the context of this clinical investigation. Indeed, the c.881C > T p.(Ala294Val) variant in *PADI3* gene is associated to Uncombable Hair Syndrome, a rare autosomal recessive disorder; thus, not only does this information have a limited clinical relevance (it just indicates the possibility that the offspring could be carrier itself), but the associated pathological condition is also not related to the motivation that induced this couple to request ECS. Similar considerations can apply also to the variant c.4981C > T p.(Arg1661Cys) in *COL4A3* gene. This gene has been related to Alport syndrome, a genetic condition with different patterns of inheritance, characterized by highly heterogeneous phenotypes, including progressive renal disease with extrarenal alterations and isolated hematuria. In particular, mutations in the *COL4A3* gene are associated to a rare autosomic recessive form of this disease. Finally, the most interesting variant is the c.809G > A p.(Arg270His) in the *LPL* gene, associated to familial lipoprotein lipase deficiency [39]. This missense variant is responsible of a defective lipase enzyme activity, that in turn causes an alteration of lipid metabolism, considering its central roles in the energy storage, metabolism, and transport of lipids [40,41]. Indeed, deficiency of this enzyme results in the accumulation of chylomicrons and an increased concentration of triglycerides in the blood. It is well known that ASD patients are lean, suggesting that the high levels of LPL activity may be involved in such clinical feature [42]. Although this phenotype has been reported in ASD mouse models [43,44,45], in adult patients with ASD also overweight and obesity have been reported [46]. Moreover, some studies investigated LPL role in brain functions: indeed, *LPL* is highly expressed in the hypothalamus, hippocampus, and striatum, and variants in this gene have been associated to neurite and Alzheimer disease. By using neuron-specific *LPL*-deficient mice, it has been demonstrated that *LPL* regulates energy balance, is important for cognitive functions and plays a crucial role in the regulation of neurons survival and differentiation in ASD [47,48]. Other studies highlighted a relationship between LPL and oxidative stress, crucial for the central nervous system (CNS) development and ASD pathophysiology [49,50,51,52]. Based on these findings, Hirai et al. hypothesized that a deregulation of LPL-associated processes may cause immature brain development and neurodevelopmental disorders, such as ASD [53]. In addition to the increased LPL activity detected in ASD [53], a study of dyslipidemia in ASD identified sex-differentially expressed, neurodevelopmentally co-regulated, ASD segregating deleterious *LPL* variants [54]. Taken together, all these findings suggest that *LPL* has to be considered as an autism candidate gene [55]. Thus, the identification of the *LPL* pathogenic variant in the male partner of this couple appears to be of particular interest considering his positive family history for ASD and that this was the motivation for ECS in this couple. In fact, driven by the desire to have a child and aware of the male partner’s family history of ASD, the couple spontaneously underwent genetic counseling. The geneticist, not having molecular information on the ASD case available, suggested that the couple carry out the ECS preconceptionally, in order to obtain an estimate of the risk of having a child affected by a genetic disease and, particularly, by ASD. Indeed, according to National guidelines [56], in a family with an ASD case, the risk of having another autistic child is 20 times greater than the general population. Moreover, ASD has a prevalence of 40/10,000 and a high rate of comorbidities, such as attention deficit hyperactivity syndrome, Tourette syndrome, obsessive compulsive disorder (DOC) and epilepsy. No previous genetic tests were referred by the couple or by the family of the male partner with the positive family history. Thus, based on the above ECS was suggested.

Respect to traditional genetic carrier screening, ECS allows to test simultaneously several genetic conditions regardless of their ethnicity [1]. Although throughout the world is growing the need for a common regulation aimed at define the timing of the test, the number of diseases included and of genes/genetic alterations analyzed, currently ECS use is still highly heterogeneous [1]. In this context, the Italian Society of Human Genetic (SIGU) suggests considering several parameters for the selection of genes to include in ECS: (i) the pathology must be associated with a well-defined phenotype; (ii) the disease must have an adverse effect on life expectation/quality of life; (iii) the disease must cause a cognitive and/or physical deficit; (iv) the disease must require medical and/or surgical intervention; (v) the disease must show an early onset; and (vi) an appropriate pre-implantation and/or prenatal diagnosis/genetic test should be available [57]. Moreover, even if ECS can be performed both contextually (when both the partners perform the test simultaneously) or sequentially (when the test is extended to the second partner in the case of a positive result in the other one), the first option should be preferred to have a better estimate regarding the couple risk of transmission of a genetic disease. Similarly, even if antenatal test is possible in the first gestational weeks, ECS should be preferably performed preconceptionally in order to allow couples autonomy in taking reproductive choices. Further studies on large number of couples are required to assess the advantages of ECS from the perspective of Health Systems costs reduction and to define the categories that are at risk and could benefit from this kind of test. Considering the analytic strategy, both targeted panels and WES analyses have been used to date for ECS purposes. Nevertheless, both of these NGS-based approaches may be inaccurate in CNVs identification requiring the integration of additional techniques. Accordingly, in the case reported herein, an integrated approach, based on both WES and aCGH, was chosen taking into account that ASD is often related to CNVs alterations. Indeed, the choice of the most proper analytic strategy should also be based on the clinical motivation for ECS.

Through this strategy, it was possible to verify the carrier status of both partners regarding the most common genetic diseases and estimate their risk of transmission, thus allowing a most proper preconception counseling to the couple. With regard to the autism risk, the ECS analysis carried out preconceptionally in this couple shows that this strategy allows the identification of variants associated with a greater susceptibility to autism, as well as hereditary genetic diseases, which would allow for better decisions on the couple’s reproductive life. Of course, the unavailability of the affected relative to undergo molecular investigations represents the main limitation of this study since it was no possible to verify the co-segregation of the *LPL* variant identified in the male partner. This possibility (i.e., finding a potentially interesting variant not being able to study it in the affected family member) was discussed with the couple during the pre-test genetic counseling and deepened at the time of the post-test genetic counseling. Moreover, even if *LPL* role in ASD pathogenesis is still under investigation and it was no possible to further study the identified variant in the context of this family, it may impair fat metabolism and the related risks were discussed by the geneticist suggesting the proper follow up tests. At the end of post-test genetic counseling, the couple understood the limits of the test discussed herein and that it was not possible to obtain a definitive result regarding their risk of having a child affected by autism. On the other hand, they were able to verify that they were not carriers of common genetic pathologies. Therefore, both partners have expressed a willingness to continue with a pregnancy and consider the possibility of a new genetic counseling after the birth of the child.

## 4. Conclusions

ECS is becoming an even more common procedure thanks to the diffusion of genetic tests and the availability of accurate molecular biology techniques allowing for the analysis of large genomic regions in a fast and time-saving manner. This approach promises to overcome limits of traditional methods, focusing on a restricted number of genes and/or causative mutations. As in the case described herein, the combination of aCGH and WES allows a better estimation of a couple’s reproductive risks. However, it has to be underlined that some concerns still limit ECS diffusion, especially with regard to the number of genes to be analyzed and the results interpretation and communication. Panels discussion between experts and scientific societies guidelines are desirable to regulate ECS supporting its correct use in clinical contexts.

## Figures and Tables

**Table 1:** Sequence data obtained by WES analysis.

Patient	Number of Sequenced Bases	Number of Reads	Number of Variants (Total)
Male partner (II.3)	1,951,404,984	33,948,962	49,517
Female partner (II.4)	1,893,654,505	32,773,550	49,548

**Table 2:** List of the potentially clinically-relevant DNA variants identified in both the partners of the analyzed couple through WES.

Chr	Gene	cDNA *	Protein *	Reference SNP ID	Status	Associated Phenotype ^†^	Clinvar Classification	ACMG/AMP ^§^ Classification
1	*MTHFR*	c.788C > T	p.Ala263Val	rs1801133	Het for both	Homocystinuria due to MTHFR deficiency-AR Neural tube defects, susceptibility to-AR Schizophrenia, susceptibility to-AD Thromboembolism, susceptibility to-AD Vascular disease, susceptibility to	Drug response	VUS
7	*PRSS1*	c.47C > T	p.Ala16Val	rs202003805	Het for both	Hereditary Pancreatitis-AD	Likely Pathogenic	Benign
11	*ACTN3*	c.1729C > T	p.Arg577Ter	rs1815739	Het for both	α-actinin-3 deficiency-AR Sprinting performance-AR	VUS	Benign
16	*IL4R*	c.223A > G	p.Ile75Val	rs1805010	Hom for him/Het for her	Atopy, susceptibility to AD	Conflicting	Benign

* Based on Human Genome Variation Society (HGVS) guidelines; ^†^ According to MedGen database; ^§^ ACMG, American College of Medical Genetics, and AMP, Association for Molecular Pathology. SNP, single nucleotide polymorphism; ID, identifier; Het, heterozygous; Hom, homozygous; AR, autosomal recessive; AD, autosomal dominant; VUS, variant of unknown significance.

**Table 3:** List of the pathogenic/likely pathogenic DNA variants identified only in the husband by WES analysis.

Chr	Gene	cDNA *	Protein *	Reference SNP ID	Status	Associated Phenotype ^†^	Clinvar Classification	ACMG/AMP ^§^ Classification
1	*PADI3*	c.881C > T	p.Ala294Val	rs144080386	Het	Uncombable hair syndrome-AR	Pathogenic	Benign
2	*COL4A3*	c.4981C > T	p.Arg1661Cys	rs201697532	Het	Alport syndrome 2-AR Alport syndrome 3-AD Hematuria-AD	Likely Pathogenic	Likely Pathogenic
8	*LPL*	c.809G > A	p.Arg270His	rs118204062	Het	Combined hyperlipidemia-AD Lipoprotein lipase deficiency-AR	Pathogenic	Pathogenic

* Based on Human Genome Variation Society (HGVS) guidelines; ^†^ According to MedGen database; ^§^ ACMG, American College of Medical Genetics, and AMP, Association for Molecular Pathology. SNP, single nucleotide polymorphism; ID, identifier; Het, heterozygous; AR, autosomal recessive; AD, autosomal dominant.

## Data Availability

Data are contained within the text. Further details can be required to corresponding authors.
